# Do conventional intracerebral hemorrhage functional scores apply to mild-to-moderate hemorrhage?

**DOI:** 10.3389/fneur.2026.1824565

**Published:** 2026-08-12

**Authors:** Ao Chen, Jiaying Peng, Liang Guo, Renhui Zhou

**Affiliations:** 1 Department of Neurosurgery, YueYang People’s Hospital, Yueyang, Hunan, China; 2 Department of Infectious Diseases, YueYang People’s Hospital, Yueyang, Hunan, China

**Keywords:** intracerebral hemorrhage, mild-to-moderate, nomogram, prediction model, risk factors

## Abstract

**Background:**

Conventional intracerebral hemorrhage (ICH) functional scores are typically developed based on clinical factors for assessing mortality risk, and their applicability in evaluating functional outcomes in patients with the specific hemorrhagic subtype of mild to moderate ICH(MTM-ICH) remains unclear. Therefore, we aimed to construct a nomogram model to predict early functional dependence in this patient population and to validate three previous general ICH functional scores.

**Methods:**

We conducted a retrospective analysis of data from 575 patients with MTM-ICH (GCS score ≥ 9) treated at our hospital between January 2018 and July 2025. The baseline demographic information, clinical characteristics, and clinical outcomes were collected. Early functional dependence was defined as a modified Rankin Scale (MRS) score ≥ 3 at discharge or 30 days after ICH. Least absolute shrinkage and selection operator (LASSO) and stepwise logistic regression analyses were used to screen for risk factors for early functional dependence and construct a predictive model. The model was visualized using a nomogram, and its predictive performance was compared with three existing ICH functional scores (FUNC, ICH-GS and ICH-FOS). Internal validation of the nomogram was performed using a 10-fold cross-validation combined with bootstrap resampling.

**Results:**

Among 575 patients with MTM-ICH, 198 (34.4%) developed early functional dependence. LASSO and stepwise logistic regression analyses identified six independent predictors: age, admission GCS score, hematoma volume, thalamic hemorrhage, basal ganglia hemorrhage, and mechanical ventilation. The nomogram based on these factors demonstrated good calibration and superior predictive performance compared to three existing ICH functional scores (AUC = 0.856 for our nomogram, 0.579 for FUNC, 0.601 for ICH-GS, and 0.714 for ICH-FOS, respectively). The decision curve analysis further demonstrated that the model had greater net benefit than the three previous general ICH functional scores. Internal validation confirmed the model’s stability and generalizability (10-fold cross-validation mean: 0.846; bootstrap-corrected C-index: 0.844).

**Conclusion:**

Among patients with MTM-ICH, advanced age, lower admission GCS score, larger hematoma volume, thalamic or basal ganglia hemorrhage, and mechanical ventilation were associated with a higher risk of early functional dependence. The nomogram developed based on these factors demonstrated high predictive performance. In contrast, conventional ICH functional scores demonstrate certain limitations when assessing this specific hemorrhage subtype.

## Introduction

Intracerebral hemorrhage (ICH) is one of the most severe and challenging types of stroke that often leads to significant disability in survivors (1). The early mortality rate ranges from 30 to 40%, and approximately 70–80% of survivors experience varying degrees of disability (2–4). Although the results of several large-scale clinical trials of ICH suggest that aggressive treatment may reduce mortality, functional outcomes have not shown substantial improvement (2, 5–10). Therefore, identifying and preventing independent risk factors for poor prognosis in ICH is critically important in clinical practice, as this may fundamentally alter clinical outcomes.

Several scoring systems have been developed to assist clinicians in predicting the outcomes of acute ICH (11–19). Most of conventional ICH functional prediction models were typically developed using clinical factors that assess mortality risk (12, 13, 15).Additionally, conventional ICH functional scores such as FUNC, ICH-GS, and ICH-FOS were originally derived from cohorts spanning the full spectrum of ICH severity, and their predictor weights are optimized for distinguishing extreme outcomes such as deep coma or massive hematoma. However, when applied to the more homogeneous MTM-ICH population, these models may suffer from reduced discriminative ability. However, while severe ICH is typically associated with increased mortality or severe disability (11), the clinical factors that lead to early functional dependence in patients with mild-to-moderate intracerebral hemorrhage (MTM-ICH) remain unclear. Consciousness level at admission and hematoma volume have consistently been regarded as the most stable prognostic predictors of ICH in previously developed models (11, 12, 19). This reflects a general bias in these models toward assigning greater weight to specific factors, such as low GCS scores or large hematoma volumes. Therefore, existing prediction models may not be suitable for the functional assessment of patients with MTM-ICH. Thus, there is a need to develop a new prediction model that enables risk stratification based on hemorrhage severity to achieve precise prognosis prediction.

In this study, we aimed to develop and validate a risk prediction model for estimating the risk of early functional dependence in patients with MTM-ICH (GCS ≥ 9). In addition, we compared the predictive ability of our model with that of three commonly used ICH functional scores (13, 15).

## Methods

### Patient population

**Figure 1:**
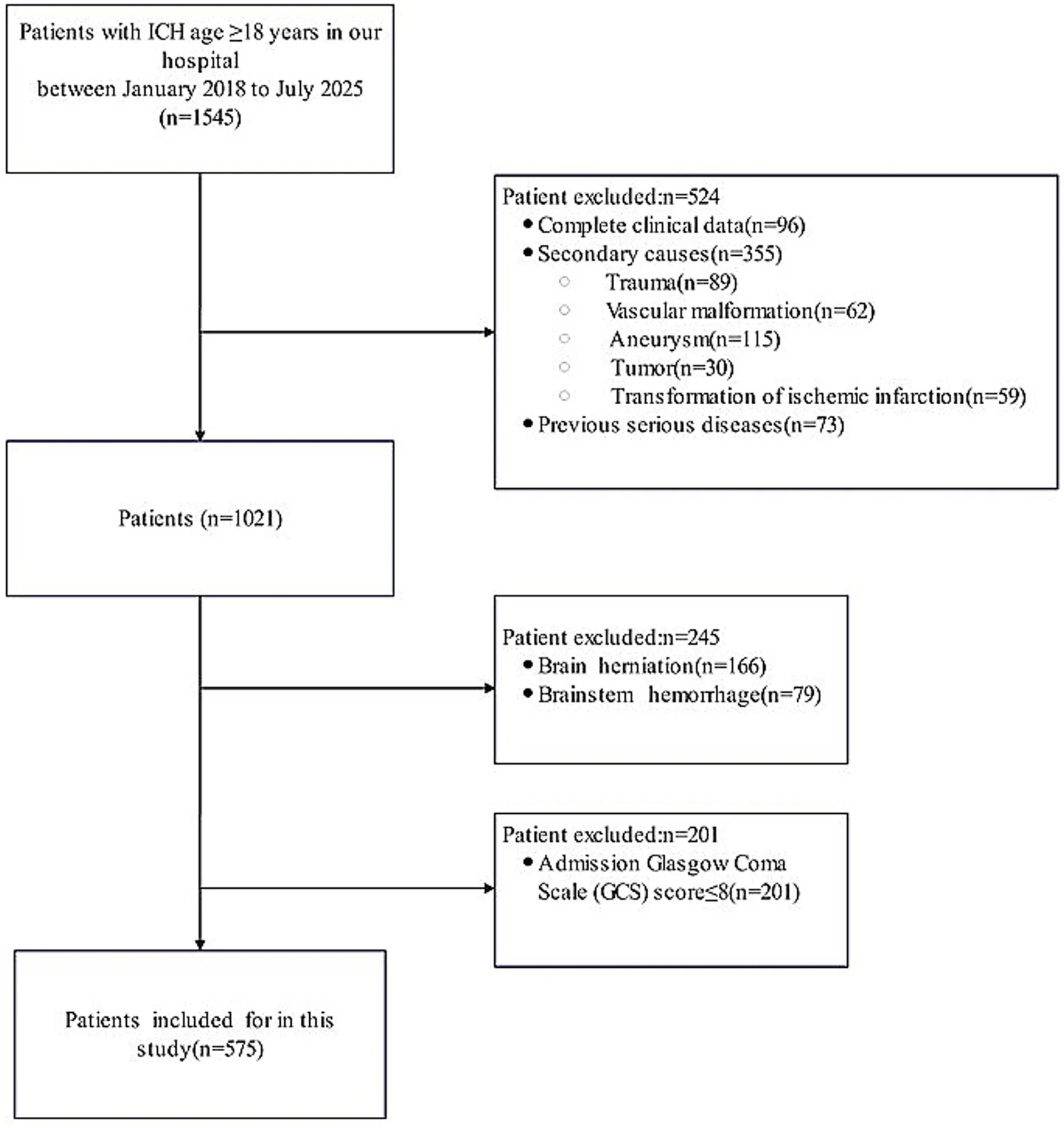
Flowchart summarizing the characteristics of patients included in the study. ICH, intracerebral hemorrhage.

This study adhered to the guidelines of the Ethics Committee and was approved by Yueyang People’s Hospital. This study was conducted in accordance with the principles of the Declaration of Helsinki. We retrospectively analyzed data from 1,545 patients with spontaneous ICH prospectively collected at our institution between January 2018 and July 2025. Among them, 575 patients met the study criteria for MTM-ICH (Figure 1).

Inclusion criteria were: age ≥ 18 years; spontaneous ICH confirmed using computed tomography or magnetic resonance imaging; and admission GCS score ≥ 9. A GCS score ≥ 9 typically indicates preserved consciousness without the need for urgent airway intervention, reflecting a fundamental difference in management strategies and prognosis compared to severe ICH (GCS ≤ 8) (1). This definition aligns with the clinical characteristics of non-surgical or conservatively managed patient populations described in previous studies (20). Exclusion criteria were patients with brain herniation (radiologically defined as midline shift ≥ 5 mm or basal cistern compression; clinically defined as deep coma, unilateral or bilateral pupillary dilation, or unstable vital signs), brainstem hemorrhage, secondary causes of hemorrhage (e.g., aneurysm, vascular malformation, tumor, hemorrhagic transformation of ischemic infarction, or traumatic injury), and severe physical or mental illnesses that can lead to significant functional impairments or other life-threatening comorbidities. Patients with brain herniation or brainstem hemorrhage were excluded because these conditions typically represent the most fatal forms of ICH, irrespective of hematoma volume, and are indicative of end-stage disease, which falls outside the scope of MTM-ICH.

### Data processing

Patients were divided into two groups: the early functional dependence group and the functional independence group. Baseline data were collected, including demographic information and medical history. Laboratory parameters included admission glucose level, body temperature, systolic blood pressure, diastolic blood pressure, white blood cell count, hemoglobin level, and platelet count. The imaging data included hemorrhage location, hematoma volume, intraventricular extension of the hematoma, and ventricular enlargement. The GCS score at admission was assessed according to standard guidelines during the onset of ICH and served as a baseline indicator of the patient’s condition. The early mechanical ventilation was defined as endotracheal intubation with invasive ventilatory support that was initiated at admission or within the first 72 h after ICH onset.

The diagnosis of ventricular enlargement was based on any of the following criteria (21, 22): (1) Evans index (maximum frontal horn width divided by maximum cranial diameter on the same plane) > 0.3; (2) maximum frontal horn width > 45 mm; (3) ventricular diameter > 6 mm; (4) ventricular volume > 12 mm. The hematoma volume was calculated using the formula (1/2 × A × B × C), where A, B, and C represent the maximum measured width, length, and height, respectively. The FUNC, ICH-GS, and ICH-FOS scores were calculated according to the original publications (12, 13, 15). Detailed scoring formulas are provided in Supplementary material.

The modified Rankin Scale (mRS) was used to assess functional recovery at discharge or 30 days after ICH in patients with mild-to-moderate ICH. Functional dependence was defined as an mRS score ≥ 3.

### Statistical analysis

Statistical analyses were performed using SPSS (version 25.0; IBM Corp., Armonk, NY, USA) and R software (version 4.1.0; R Foundation for Statistical Computing, Vienna, Austria). Quantitative variables were expressed as median (25th–75th percentiles) or mean (± standard deviation), while categorical variables were presented as percentages and frequencies. Univariate analysis identified the following risk factors associated with early functional dependence: hypertension, admission GCS score, intraventricular hematoma extension, hematoma volume, basal ganglia hemorrhage, lobar hemorrhage, hematoma evacuation, ICU admission, mechanical ventilation, and pneumonia.

Variable selection was further performed using LASSO regression and stepwise logistic regression analysis to establish the prediction equation. A nomogram was constructed based on the contribution of each independent risk factor to the outcome variables. Considering the predefined hematoma volume criteria upon admission, we developed a graphical model named the “MTM-ICH” probability chart.

The discriminative performance of the model was evaluated using receiver operating characteristic (ROC) curve analysis, and calibrated using calibration plots and the Hosmer–Lemeshow test. The clinical utility of the prediction model was examined using decision curve analysis (DCA). The predictive capability and clinical applicability of the MTM-ICH probability chart for early functional dependence in MTM-ICH were compared with three existing ICH functional scores (FUNC, ICH-GS and ICH-FOS) by analyzing the area under the ROC curve (AUC) and DCA values. Internal validation was conducted using 10-fold cross-validation combined with 1,000 Bootstrap resampling iterations. A close agreement between the C-indices derived from both methods indicated good model stability and generalizability. Statistical significance was set at *p* < 0.05.

## Results

As shown in Figure 1, 575 patients with ICH met the inclusion criteria and were enrolled in the study. Table 1 presents the univariate analysis results of their baseline characteristics. Among them, 198 patients (34.4%) were classified into the early functional dependence group, while the remaining 377 (65.6%) were assigned to the early functional independence group.

**Table 1:** The baseline characteristics of the patients with MTM-ICH.

Variables	Overall (*n* = 575)	Functional independence (*n* = 377)	Functional dependence (*n* = 198)	*P* value
Age, median (IQR)	65 (56, 74)	65 (56, 73)	67 (57, 74)	0.115
Male [*n* (%)]	409 (71)	273 (72)	136 (69)	0.401
Medical history				
Hypertension	437 (76)	272 (72)	165 (83)	0.004
Diabetes	72 (12)	45 (12)	26 (13)	0.779
History of stroke Coronary heart disease	26 (5) 72 (13)	13 (3) 49 (13)	13 (7) 23 (12)	0.134 0.732
Admission clinical features (IQR)				
Temperature, °C	36.5 (36.5, 36.6)	36.5 (36.5, 36.6)	36.5 (36.5, 36.6)	0.529
Systolic BP, mmHg	163 (147, 181)	163 (146, 183)	164 (141, 185)	0.822
Diastolic BP, mmHg	89 (75, 102)	92 (78, 101)	90 (76, 101)	0.452
Admission GCS score (IQR)	14 (12, 15)	15 (13, 15)	12 (10, 14)	<0.001
9-12 [*n* (%)]	170 (30)	63 (17)	107 (54)	<0.001
13-15 [*n* (%)]	405 (70)	312 (83)	93 (46)	<0.001
Radiological data [*n* (%)]				
Hematoma extending to ventricles	146 (25)	83 (22)	63 (32)	0.014
Total volume, ml	9 (3, 15)	6 (3, 12)	15 (9, 22)	<0.001
Cerebellar hemorrhage	65 (11)	45 (12)	20 (10)	0.602
Thalamic hemorrhage	145 (25)	95 (25)	50 (25)	0.999
Basal ganglia hemorrhage	241 (42)	142 (38)	99 (50)	0.001
Lobar hemorrhage	124 (22)	95 (25)	29 (15)	0.015
Ventricular enlargement	9 (2)	4 (1)	5 (3)	0.287
Treatment [*n* (%)]				
Surgical hematoma evacuation	94 (16)	31 (8)	63 (32)	<0.001
EVD	41 (7)	22 (6)	19 (10)	0.135
ICU admission	83 (14)	35 (9)	48 (24)	<0.001
Mechanical ventilation	81 (14)	23 (6)	58 (30)	<0.001
Pneumonia	290 (50)	149 (40)	141 (71)	<0.001
Laboratory data (IQR)				
Serum glucose	6.3 (5.7, 7.5)	6.2 (5.7, 7.5)	6.4 (5.7, 7.7)	0.591
WBC, 10^9^/L	8.3 (6.2, 11.1)	8.2 (6.3, 11.5)	8.3 (6.1, 10.7)	0.325
PLT, 10^9^ /L	170 (142, 213)	168 (139, 212)	178 (149, 216)	0.062
Hemoglobin, g/L	141 (125, 152)	141 (126, 150)	141 (128, 150)	0.655

GCS, Glasgow coma score; BP, blood pressure; EVD, external ventricular drainage; LD, Lumbar drainage; WBC: white blood cell; PLT, blood platelet. Values are shown as (IQR), or *n* (%).

Compared to the early functional independence group, patients in the early functional dependence group had a higher prevalence of hypertension (83% vs. 72%; *p* = 0.004), lower admission GCS scores [12 (IQR 10–14) vs. 15 (IQR 13–15); *p* < 0.001], larger hematoma volumes [15 (IQR 9–22) mL vs. 6 (IQR 3–12) mL; *p* < 0.001], higher rates of hematoma extending to ventricles (32% vs. 22%; *p* = 0.014), and a greater likelihood of basal ganglia hemorrhage (50% vs. 38%; *p* < 0.001) and a lower likelihood of lobar hemorrhage (15% vs. 25%; *p* = 0.015).

Additionally, patients with early functional dependence had higher rates of ICU admission (24% vs. 9%; *p* < 0.001), mechanical ventilation (30% vs. 6%; *p* < 0.001), pneumonia (71% vs. 40%; *p* < 0.001), and hematoma evacuation (32% vs. 8%; *p* < 0.001). No significant associations were found between early functional dependence and other laboratory or clinical parameters.

### Nomogram model

**Figure 2:**
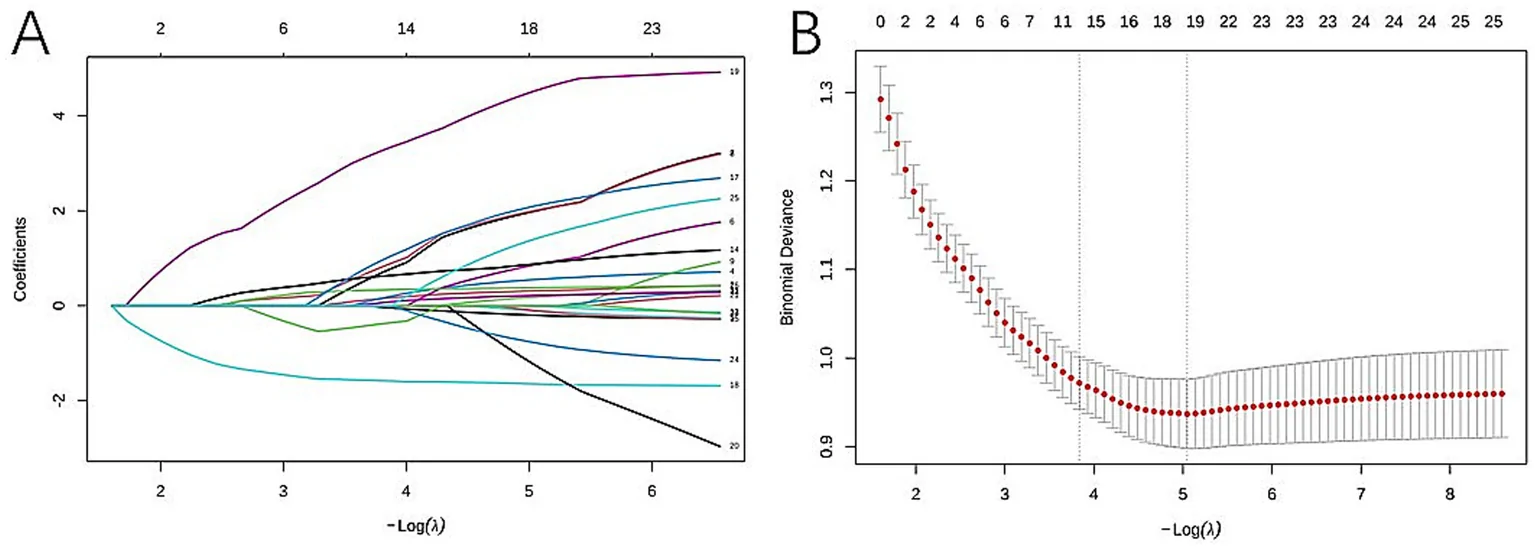
Feature selection using the least absolute shrinkage and selection operator (LASSO) binary logistic regression model. **(A)** LASSO coefficient profiles of the 27 candidate features. The coefficient profile is plotted against the log(*λ*) sequence. A vertical dashed line is drawn at the *λ* value selected using 10-fold cross-validation (lambda.min), where the optimal *λ* yielded 12 features with non-zero coefficients. **(B)** Binomial deviance curve plotted against log(*λ*). The left vertical dashed line indicates lambda.min (the minimum cross-validated deviance), and the right dotted vertical line indicates lambda.1se (one standard error from the minimum). The final model was constructed using the 12 features selected at lambda.min.

**Figure 3:**
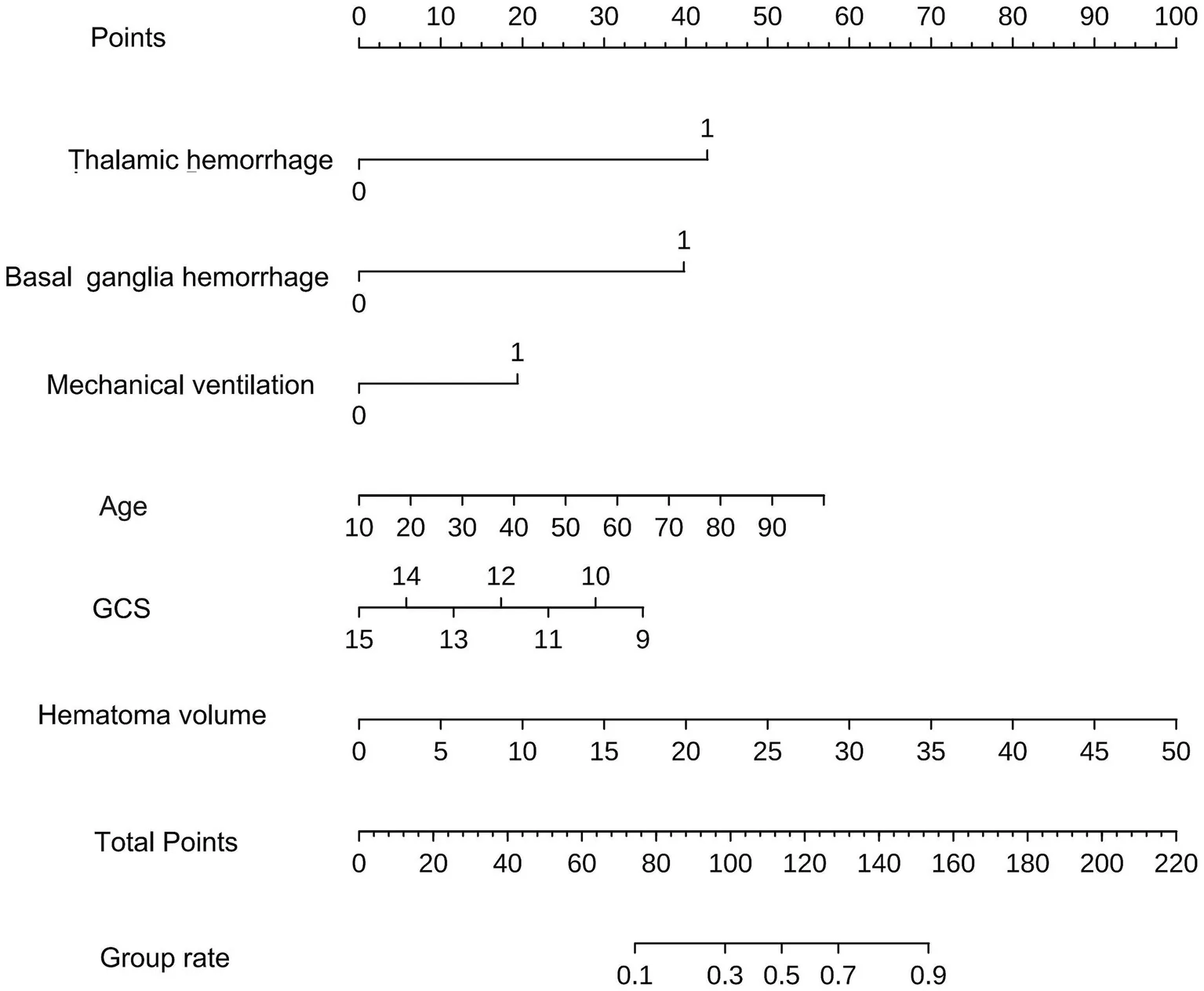
Nomogram for the prediction of early functional dependence in patients with MTM-ICH. Lines 2–7 show the six predictive variables in the equation: age, admission GCS score, hematoma volume, thalamic hemorrhage, basal ganglia hemorrhage, and mechanical ventilation. 0 indicates no, 1 indicates yes. GCS, Glasgow Coma Scale; MTM-ICH, mild-to-moderate intracerebral hemorrhage.

LASSO regression was applied to select variables from clinical, laboratory, and imaging data of 575 patients with MTM-ICH. Among 27 initial features, 12 potential predictors were retained, including sex, age, history of hypertension, history of stroke, GCS score, hematoma volume, thalamic hemorrhage, basal ganglia hemorrhage, lobar hemorrhage, hematoma evacuation, mechanical ventilation, and pneumonia (Figure 2). Stepwise logistic regression analysis identified six independent predictors of early functional dependence: age, admission GCS score, hematoma volume, thalamic hemorrhage, basal ganglia hemorrhage, and mechanical ventilation (Table 2). A nomogram incorporating these six independent risk factors was developed (Figure 3), with higher total scores indicating a greater likelihood of early functional dependence. The complete regression equation and detailed model performance metrics are provided in the Supplementary material.

**Table 2:** Prediction factors for early functional dependence in MTM-ICH.

Variable	*β*	OR (95% CI)	*P* value
Intercept	-0.184	0.157 (0.014-1.668)	0.129
Thalamic hemorrhage	2.369	10.689 (5.067-23.768)	<0.001
Basal ganglia hemorrhage	2.210	9.118 (4.909-17.817)	<0.001
Mechanical ventilation	1.077	2.937 (1.519-5.782)	<0.001
GCS score	-0.322	0.725 (0.640-0.819)	<0.001
Age	0.035	1.036 (1.017-1.056)	<0.001
Hematoma volume	0.111	1.118 (1.082-1.157)	<0.001

### Model evaluation

**Figure 4:**
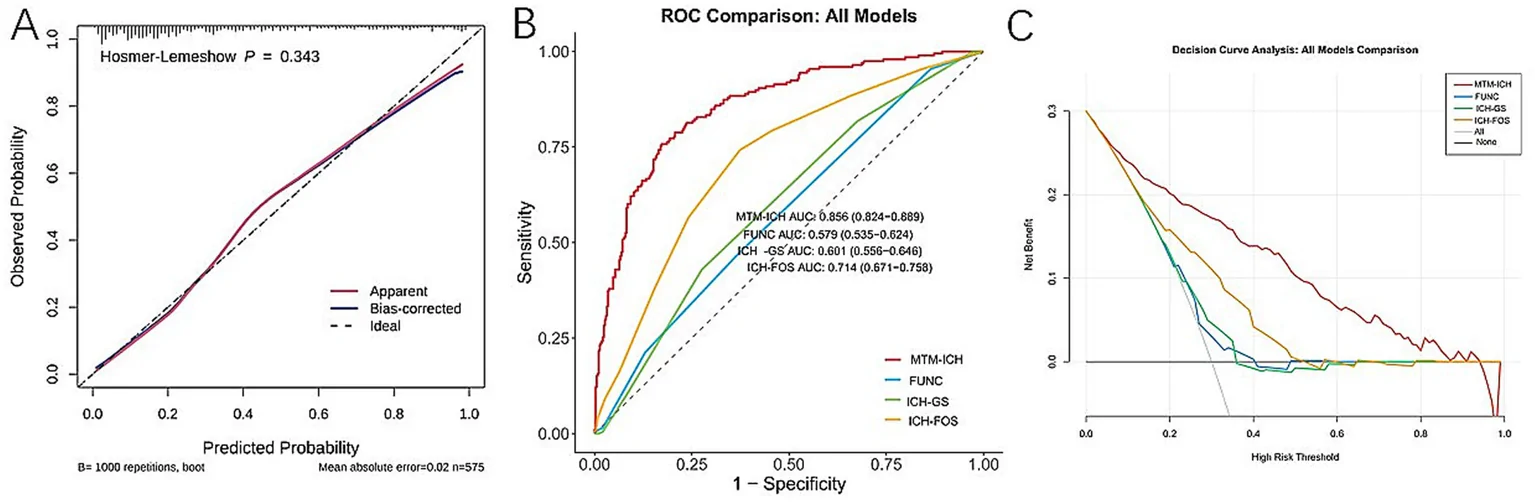
Model evaluation. **(A)** Calibration plot for nomogram-predicted probability of early functional dependence. **(B)** Receiver operating characteristic curves detect the discriminative ability of our nomogram and FUNC score, ICH-GS score, and ICH-FOS score in the cohort. **(C)** Decision curve analysis of our nomogram and three general ICH score in the cohort. AUC: Area under the curve; ICH: Intracranial hemorrhage.

**Figure 5:**
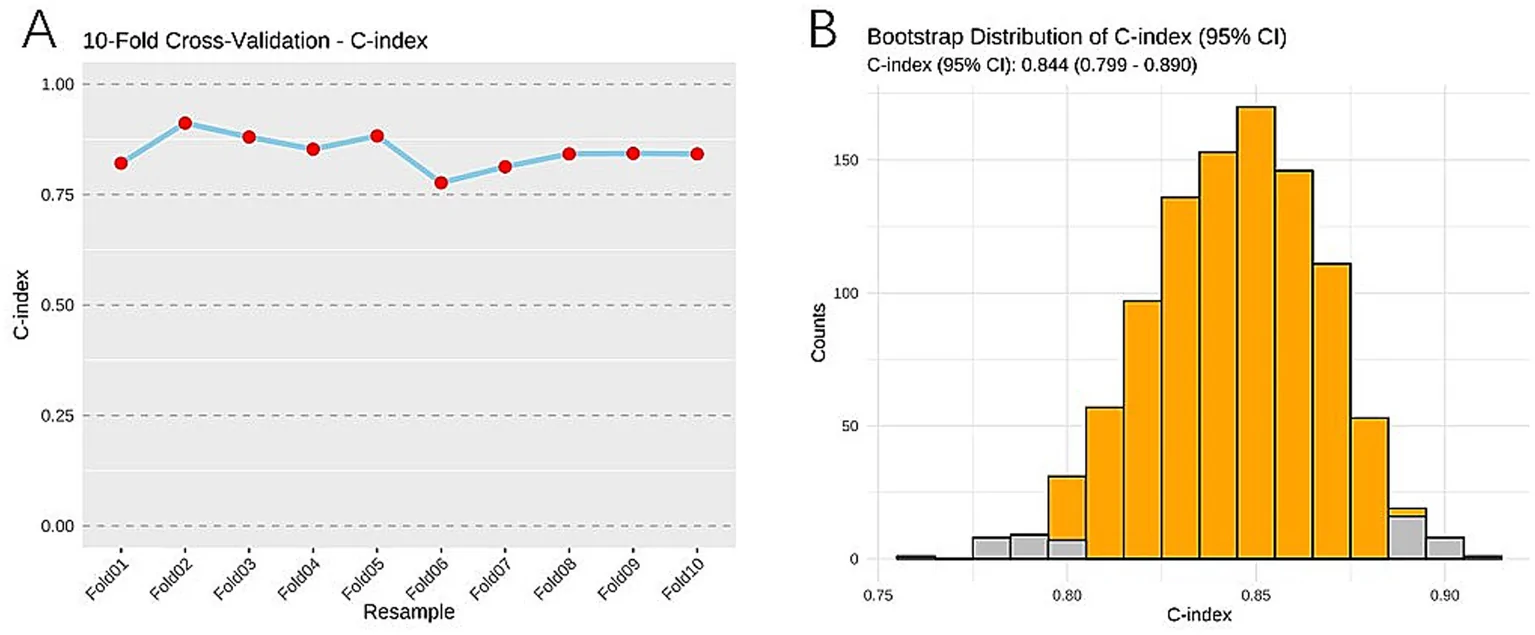
Internal validation of the nomogram model. **(A)** The average C-index obtained from 10-fold cross-validation for internal validation is 0.846. **(B)** The C-index obtained after 1,000 iterations of bootstrap sampling is 0.844 (95% CI: 0.799–0.890). CI, confidence interval.

The calibration plot demonstrated excellent agreement between predicted and observed probabilities (mean absolute error = 0.019) (Figure 4). The ROC curve analysis revealed an AUC of 0. 856 [95% confidence interval (CI): 0.824–0.889 for predicting early functional dependence, indicating strong discriminative ability and significantly outperforming the FUNC score (AUC = 0.579; 95% CI: 0.535–0.624)], the ICH-GS score (AUC = 0.601; 95% CI: 0.556–0.646) and the ICH-FOS score (AUC = 0.714; 95% CI: 0.671–0.758) (Figure 4). Internal validation using 10-fold cross-validation and 1,000 bootstrap resampling iterations yielded C-indices of 0.846 and 0.844 (95% CI: 0.799–0.89), respectively, which were close to the AUC values obtained from the ROC analysis, confirming the model’s stability and generalizability (Figure 5). Finally, DCA demonstrated significant net clinical benefit, surpassing that of the traditional three ICH functional scores and highlighting the model’s strong clinical utility (Figure 4).

## Discussion

Over the past two decades, numerous predictive models have been developed for ICH, most of which are derived from the general ICH population (11–19). Models predicting functional outcomes often resemble those for mortality, utilizing similar clinical predictors (12, 13, 15) frequently automatically assigning greater weight to factors such as large hematoma volume or low consciousness levels (11, 12, 14–17, 19). This approach may introduce a systematic bias that limits their accuracy in predicting functional outcomes, particularly in patients with mild or moderate ICH. The GCS score serves as a simple yet clinically important indicator for assessing initial severity in patients with ICH, and a score ≥ 9 has been widely adopted in previous studies to define mild to moderate cases (1, 20, 23, 24). Compared to patients with severe ICH, the factors driving early poor functional outcomes in mild to moderate ICH remain poorly understood. Therefore, further risk stratification based on hemorrhage severity is essential to identify independent predictors of functional dependence in this patient subgroup and inform targeted clinical decision-making.

In the INTERACT2 trial, which included 2,839 patients (median GCS score, 14) (6), those with GCS scores of 3–5 and those who underwent surgical interventions were excluded. The incidence of the primary outcome (mRS score, 3–6) at 90 days was 52.0 and 55.6% in the intensive blood pressure-lowering and standard treatment groups, respectively. In the INTERACT3 trial, which enrolled 7,036 patients (median GCS score, 12) (7), the corresponding risks were 60.1 and 63.2% in the intervention and control groups, respectively. Notably, INTERACT3 included patients who underwent surgical treatment. In our cohort of patients with MTM-ICH (GCS ≥ 9; median GCS score, 14), the incidence of early functional dependence (mRS score, 3–6) was 34.4% (198/575), which appears significantly lower than the rates observed in the two aforementioned clinical trials. This difference may be attributed to our stricter inclusion criteria regarding admission consciousness level. Although our study was based on early post-ICH assessment and did not account for subsequent aggressive rehabilitation and nursing interventions, this finding suggests that variations in consciousness levels at admission may lead to divergent prognostic outcomes in patients with ICH.

Admission consciousness level and hematoma volume have consistently been regarded among the most stable predictors of ICH outcomes in prior research (11, 12, 19). A previous study comparing 19 existing ICH scores found that the National Institutes of Health Stroke Scale alone could effectively quantify in-hospital mortality risk (18). Another study evaluated the performance of the original ICH score, ICH grading scale, and modified ICH score in predicting 30-day mortality, concluding that all three scores performed similarly and suggesting that the simplest measure, the GCS score, may suffice for prognostication after ICH (25). In our developed prediction model, despite restricting the admission GCS scores, we found that both GCS and hematoma volume can still serve as independent risk predictors for functional outcomes in patients with mild to moderate ICH. This reaffirms the stability of admission consciousness level and hematoma volume in prognostic assessment across all hemorrhage types.

Deep hemorrhages, including those of the thalamus and basal ganglia, are closely associated with functional dependence. Similarly, our study identified these locations as strong independent predictors of early functional dependence in patients with MTM-ICH. Both the STICH I and STICH II trials (9, 10) demonstrated that for spontaneous supratentorial ICH (most of which are deep hemorrhages), early surgical hematoma evacuation did not significantly improve functional outcomes compared with initial medical management. This strongly suggests that while surgery can relieve mechanical compression from the hematoma, it cannot reverse the damage caused by hemorrhage to critical neural fibers and nuclei, even in patients with mild-to-moderate ICH who receive aggressive clinical treatment in our study. A post-hoc analysis of the INTERACT2 trial (26) further revealed that elderly patients with acute ICH had worse outcomes than younger patients, including higher rates of death, functional dependence, and reduced quality of life. When other confounding factors were considered, stroke patients aged > 75 years were four times more likely to die or experience disability within 90 days than those aged < 52 years. Our model also indicates a significant association between advanced age and early functional dependence, even in patients with mild-to-moderate ICH.

Deterioration of respiratory, circulatory, or neurological conditions may necessitate intensive care for patients with ICH, because pathophysiological changes can interact across organ systems (27). Brain injury induced by hemorrhage can trigger an inflammatory immune response, and sustained inflammation may suppress systemic and cellular immune responses, ultimately increasing the risk of stroke-associated pneumonia. Similarly, persistent hypoxia caused by severe pneumonia can exacerbate brain damage (28). Many existing general ICH models do not adequately consider the effect of these dynamic clinical indicators on ICH prognosis. However, our developed model revealed that mechanical ventilation was a significant predictor of early functional dependence in patients with ICH. Although an admission GCS score ≥ 9 typically indicates no need for urgent airway intervention, the requirement for mechanical ventilation often signals worsening neurological function, severe respiratory complications, or circulatory failure after admission. However, compared to simple intensive care unit admission or diagnosis of pulmonary infection, mechanical ventilation represents a more objective and severe endpoint.

When the three existing ICH functional scores (FUNC, ICH-GS, and ICH-FOS) were applied to our cohort, we found their discriminative ability to be significantly limited (AUCs of 0.58, 0.60, and 0.71, respectively), whereas our model demonstrated superior performance (AUC = 0.86). We believe this discrepancy stems from two fundamental reasons. First, the predictive weights used in the general scores (such as heavy reliance on very low GCS and large hematoma volume) become ineffective in the relatively homogeneous MTM-ICH population (GCS ≥ 9 and limited hematoma volume) due to compressed variable ranges. Second, and more critically, the drivers of a functional independence outcome are fundamentally different from those of a mortality outcome. The former depends not only on basic consciousness level and hematoma volume but is also strongly associated with injuries to specific anatomical locations and systemic complications during the clinical course. Our study confirms that within this specific MTM-ICH subtype, the architecture of general prognostic models developed based on mortality risk is not fully applicable for predicting functional outcomes.

Our study has several limitations that should be acknowledged. First, as a single-center retrospective study with a relatively long study period, temporal bias cannot be entirely excluded. Furthermore, inherent selection bias and unmeasured confounding remain inherent limitations. Although we adjusted for known confounding factors to the greatest possible extent. Although we adjusted for known confounding factors to the greatest possible extent, the potential influence of unmeasured variables (such as incompletely documented laboratory or clinical parameters) on the results cannot be excluded. Second, the primary outcome was defined as mRS ≥ 3 at discharge or 30 days after ICH onset. Although discharge timing may vary, this composite endpoint was chosen to maximize outcome ascertainment in this retrospective cohort. Finally, our predictive model lacked external validation and requires confirmation in future multicenter prospective studies.

## Conclusion

Our findings indicate that conventional ICH functional prognosis models are not applicable for predicting functional outcomes in mild-to-moderate ICH patients, as they are typically developed based on mortality risk factors. Furthermore, we developed and validated a prognostic nomogram specifically for patients with MTM-ICH (GCS ≥ 9). The model incorporated six robust independent predictors: age, admission GCS score, hematoma volume, thalamic hemorrhage, basal ganglia hemorrhage, and mechanical ventilation. It demonstrated excellent discriminative ability, significantly outperforming the existing ICH functional scoring systems (FUNC, ICH-GS and ICH-FOS). This nomogram can serve as an intuitive tool to help clinicians rapidly identify high-risk patients with MTM-ICH, enabling earlier initiation of intensive monitoring and personalized rehabilitation strategies to improve functional outcomes.

## Data Availability

The original contributions presented in the study are included in the article/Supplementary material, further inquiries can be directed to the corresponding author/s.
