# Supplementary material for: Do conventional intracerebral hemorrhage functional scores apply to mild-to-moderate hemorrhage?
Source: Front Neurol. 2026 Aug 12;17:1824565. doi: 10.3389/fneur.2026.1824565 (PMC13506375; doi:10.3389/fneur.2026.1824565)
Supplement: Supplementary file 1 [file Supplementary_file_1.docx]

**Supplementary Table S1.** The ICH-Functional Outcome Score (ICH-FOS).

| Items | Score |
| --- | --- |
| **Age group** | 0 |
| ≤59 | 1 |
| 60 to 69 | 2 |
| 70 to 79 | 4 |
| ≥80 |  |
| **Admission NIHSS score** |  |
| 0 to 5 | 0 |
| 6 to 10 | 2 |
| 11 to 15 | 3 |
| 16 to 20 | 4 |
| ≥21 | 5 |
| **Admission GCS score** |  |
| 15 to 13 | 0 |
| 9 to 12 | 1 |
| 3 to 8 | 2 |
| **Admission glucose (mmol/L)** |  |
| ≤11.0 | 0 |
| ≥11.1 | 1 |
| **ICH location** |  |
| Supratentorial | 0 |
| Infratentorial | 1 |
| **ICH volume** |  |
| For supratentorial location |  |
| <40 ml | 0 |
| 40 to 70 ml | 2 |
| >70 ml | 2 |
| For infratentorial location |  |
| <10 ml | 0 |
| 10 to 20 ml | 2 |
| >20 ml | 2 |
| **Extension into ventricles** |  |
| No | 0 |
| Yes | 1 |

**Supplementary Table S2.** The ICH Grading Scale (ICH-GS).

| Characteristic | Points |
| --- | --- |
| **Age, years** |  |
| <45 years | 1 |
| 45–64 years | 2 |
| >65 years | 3 |
| **GCS score** |  |
| 13–15 | 1 |
| 9–12 | 2 |
| 3–8 | 3 |
| **ICH location** |  |
| Supratentorial | 1 |
| Infratentorial | 2 |
| **ICH volume** |  |
| For supratentorial location |  |
| <40 mL | 1 |
| 40–70 mL | 2 |
| >70 mL | 3 |
| For infratentorial location |  |
| <10 mL | 1 |
| 10–20 mL | 2 |
| >20 mL | 3 |
| **Extension into ventricles** |  |
| No | 1 |
| Yes | 2 |

**Supplementary Table S3.** The FUNC Score.

| Component | Points |
| --- | --- |
| **ICH volume** |  |
| <30 | 4 |
| 30–60 | 2 |
| >60 | 0 |
| **Age, years** |  |
| <70 | 2 |
| 70–79 | 1 |
| **≥**80 | 0 |
| **ICH location** |  |
| Lobar | 2 |
| Deep | 1 |
| Infratentorial | 0 |
| **GCS score** |  |
| **≥**9 | 2 |
| **≤**8 | 0 |
| **Pre-ICH cognitive impairment** |  |
| No | 1 |
| Yes | 0 |

**Supplementary Table S4.** Predictive performance of the nomogram at the optimal threshold.

| Metric | Value |
| --- | --- |
| Optimal cutoff | 0.3686 |
| Sensitivity | 75.8% |
| Specificity | 82.8% |
| Positive predictive value (PPV) | 69.8% |
| Negative predictive value (NPV) | 86.7% |

**Complete regression coefficients and prediction equation for the final multivariable logistic regression model.** The prediction equation is: Logit(p)= −1.849+2.369×(thalamic hemorrhage)+2.210×(basal ganglia hemorrhage) +1.077×(mechanical ventilation)+0.035×(age) − 0.322×(GCS score)+ 0.111×(hematoma volume). The probability of early functional dependence can be calculated as p = 1/(1 + e^(-Logit)).
